# Iron Stores of Breastfed Infants during the First Year of Life

**DOI:** 10.3390/nu6052023

**Published:** 2014-05-21

**Authors:** Ekhard E. Ziegler, Steven E. Nelson, Janice M. Jeter

**Affiliations:** Department of Pediatrics, University of Iowa, A136 MTF, 2501 Crosspark Rd., Coralville, IA 52241-8802, USA; E-Mails: steven-nelson@uiowa.edu (S.E.N.); janice-jeter@uiowa.edu (J.M.J.)

**Keywords:** iron endowment, breastfed infant, iron stores, iron deficiency

## Abstract

The birth iron endowment provides iron for growth in the first months of life. We describe the iron endowment under conditions of low dietary iron supply. Subjects were infants participating in a trial of Vitamin D supplementation from 1 to 9 months. Infants were exclusively breastfed at enrollment but could receive complementary foods from 4 months but not formula. Plasma ferritin (PF) and transferrin receptor (TfR) were determined at 1, 2, 4, 5.5, 7.5, 9 and 12 months. At 1 month PF ranged from 38 to 752 µg/L and was only weakly related to maternal PF. PF declined subsequently and flattened out at 5.5 months. PF of females was significantly higher than PF of males except at 12 months. TfR increased with age and was inversely correlated with PF. PF and TfR tracked strongly until 9 months. Iron deficiency (PF < 10 µg/L) began to appear at 4 months and increased in frequency until 9 months. Infants with ID were born with low iron endowment. We concluded that the birth iron endowment is highly variable in size and a small endowment places infants at risk of iron deficiency before 6 months. Boys have smaller iron endowments and are at greater risk of iron deficiency than girls.

## 1. Introduction

At birth, the body iron content of the infant is high (94 mg/kg fat-free mass) [1] due to a high hemoglobin mass and a sizable amount of storage iron [2]. This birth iron endowment renders the infant independent of exogenous iron during the early months of life. Immediately after birth, the hemoglobin mass begins to shrinks and its iron is transferred to the storage compartment [3]. The latter therefore represents the entire birth iron endowment. Plasma ferritin (PF) concentration is proportional to storage iron and thus provides a measure to follow the fate of the iron endowment. At birth, the size of the endowment varies greatly, as we [4,5,6] and others [7,8,9,10,11,12,13,14,15,16,17,18] have shown. Although severe maternal iron deficiency [11] and certain pregnancy complications [19] are known to reduce the size of the iron endowment, under most circumstances iron status of the mother is not a determinant of the size of the iron endowment. The cause(s) of the variation in its size remain largely unknown. 

The iron endowment provides iron for growth and protects the breastfed infant against iron deficiency in the first 4–6 months of life. It follows that the size of the endowment should determine the degree of protection afforded the infant. Indeed, there is evidence that diminished size of the storage iron compartment shortens the protection and places the infant at risk of iron deficiency [4,5,6,20]. Essentially, all breastfed infants in our studies [4,5,6] who developed iron deficiency by 6 months were born with diminished iron endowment. 

As the iron endowment is used up for growth, PF concentration declines, but the rate of decline is modified by exogenous iron [4,5,6]. Few data exist regarding the unmodified iron endowment. Such data may be valuable for examining longitudinal tracking of iron stores as well as defining gender-related differences of iron stores. Only one cohort of infants in our earlier studies (control group in [5]) came close to having an unmodified iron endowment, but as even these infants could receive supplemental formula, their iron endowment could have been modified to a significant extent. Therefore, when in a recently completed study [21] it was deemed necessary to prohibit the consumption of supplemental formula until 9 months of age, the opportunity to evaluate the size of the iron endowment minimally modified by dietary iron presented itself. The present report concerns data on the iron endowment of these infants who, besides breast milk, received no source of iron until 4 months and thereafter received iron only from complementary foods until 9 months. For this large group of breastfed infants, iron status of the mothers soon after birth was known and detailed dietary information was available.

We define iron deficiency (ID) as a state of exhausted iron stores indicated by PF < 10 µg/L. When there is, in addition, evidence of impaired hemoglobin synthesis, iron deficiency is considered to be severe. Iron-deficiency anemia (IDA) carries a significant risk of impaired neurocognitive development [22,23].

## 2. Experimental Section

Clinical Trial Registration: Registered at clinicaltrials.gov NCT00494104.

Briefly, the parent study [21] was a randomized double-blind trial of breastfed infants who received one of four doses of Vitamin D supplementation (200 IU/day, 400 IU/day, 600 IU/day and 800 IU/day) from 1 to 9 months. Plasma 25(OH)D concentration was the primary endpoint. In order to minimize dietary Vitamin D intake, parents were asked not to feed supplemental formula. 

Study design and intervals: The study was a prospective, randomized, double-blind study. Infants were enrolled and randomized at 1 month (=within 4 days of 28 days). They visited the study center every 28 days until 9 months (280 ± 4 days) and made a final visit at 12 months (364 ± 4 days). Infants received the study Vitamin D drops from 1 to 9 months. The study protocol was reviewed and approved by the University of Iowa Institutional Review Board and parents provided written consent. The trial was registered with ClinicalTrials.gov under NCT00494104. 

Subjects were full-term infants considered normal by their parents and their physicians. Infants were exclusively breastfed at enrollment. Starting at 4 months, they could receive complementary foods but no formula until 9 months. Vitamin or mineral (iron) supplements were not permitted. Infants were born between August 2006 and September 2010. Enrollment was limited to infants born between June and November so they would be between 5.5 and 9 months old at the end of winter (March to mid-May) when the primary assessment of Vitamin D status took place. Parents were asked not to give any iron or Vitamin D supplements. 

Procedures: Infants visited the study center every 28 days and had weight and length measured. Parents completed an interim health and feeding questionnaire. Code-labeled Vitamin D supplements were dispensed during visits and empty and half-empty containers were collected and weighed. Infants had blood drawn at 1, 4, 5.5, 7.5, 9 and 12 months by heel prick using a disposable spring-loaded device (Tenderfoot, International Technidyne) into heparin-coated tubes. Plasma ferritin (PF) was determined using an immunoradiometric procedure (Ramco catalog no. F-11) with interassay coefficient of variation of 6.5%. Soluble transferrin receptor (TfR) was measured by enzyme immuno assay (Ramco catalog no TF-94).

Data analysis: Iron deficiency was defined as plasma ferritin <10 µg/L [4,5,6] and anemia as hemoglobin <105 g/L before 9 months and <100 g/L at 9 and 12 months [24]. Body iron was calculated as Body iron (mg/kg) = −(log(TfR/PF) − 2.8229)/0.1207, where TfR and PF are both in µg/L [25]. Gender-related differences of PF, TfR and body iron assessed by t-tests and ANOVA procedures. Tracking was determined by linear correlations between successive values. Associations of PF and TfR were determined cross-sectionally on an age-specific basis by linear correlation analysis. Percentiles of PF were determined by SAS univariate procedure. Statistical analyses were performed using SAS version 9.1.3 (SAS Institute, Cary, NC, USA).

## 3. Results

Of 213 infants enrolled at 1 month and assigned to one of the Vitamin D supplement doses, 128 completed the intervention at 9 months and 120 were evaluated at 12 months. The main reason why infants left the study was the parents’ desire to use supplemental formula. At enrollment, 177 mothers donated a venous blood sample. Table 1 summarizes feeding data as reported by the parents and shows that parents adhered to feeding rules to a remarkable degree. Only one infant received cereal at 2 and 3 months and 2 infants received formula at 3 months, but the amounts were in each case small (≤1 feeding/week) and infants continued in the study. Many infants did not receive complementary foods until late. For example, at 5.5 months 63 infants (of 153) received no complementary foods at all. Also, cereals, a rich source of iron, were received by only one-half of infants at 5.5 months. 

**Figure 1:**
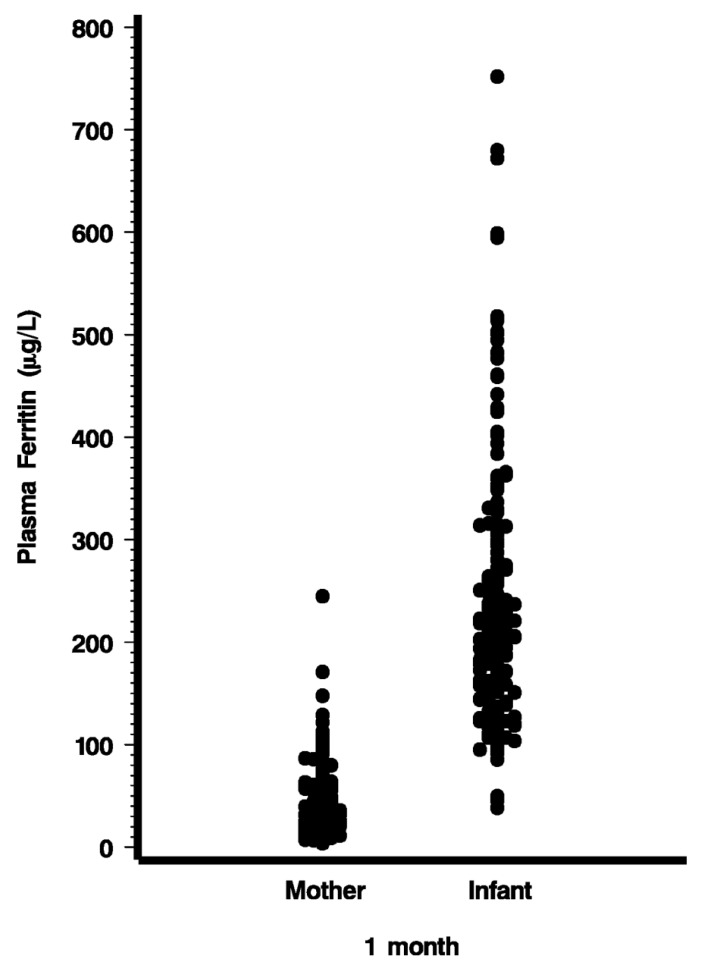
Plasma ferritin concentrations of mothers and infants one month after birth.

**Figure 2:**
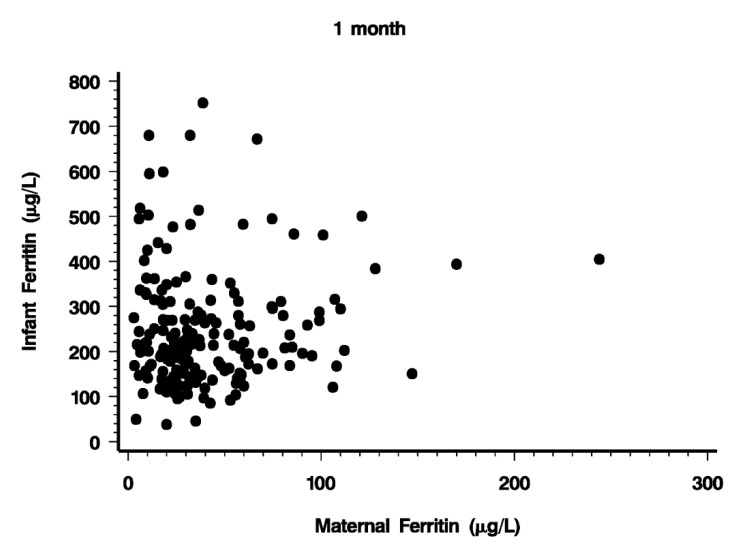
Relationship between maternal and infant plasma ferritin one month after birth (*r* = 0.081, *p* = 0.283).

At 1 month, PF averaged 242 µg/L with a range from 38 to 752 µg/L (Figure 1, Table 2). Maternal PF obtained at the same time averaged 42 µg/L (SD 34 µg/L) (Figure 1). It is evident that a fair proportion of mothers were in less than optimal iron nutritional status, with 12 mothers having PF < 10 µg/L. However, iron status of the mother was not an important factor determining infant iron stores. As illustrated in Figure 2, the relationship between maternal and infant PF levels was weak (*r* = 0.081, *p* = 0.283). Maternal iron status explained only 6.4% of the variation in infant iron endowment. As Figure 2 shows, when the mother’s iron stores were low (PF < 20 µg/L, her infant’s PF could still range from 40 to 680 µg/L.

**Table 1:** Feedings as reported by parents. The table indicates number of infants receiving the specified food during the month preceding the visit.

Age (month)	Total subjects	Any breast	Cereal	Fruits	Vegetables	Meats	Table foods	Formula	Cow milk
1	213	213	0	0	0	0	0	0	0
2	194	194	1	0	0	0	0	0	0
3	181	181	1	0	0	0	0	2	0
4	165	165	4	2	0	0	0	1	0
5.5	153	152	76	29	40	0	5	1	0
7.5	138	138	79	92	97	12	20	4	1
9	128	124	86	96	99	30	46	9	1
12	120	92	43	70	69	44	92	23	43

**Table 2:** Plasma concentrations of ferritin (PF) and transferrin receptor (TfR). Values are mean ± SD unless otherwise indicated.

Age (month)	1	2	4	5.5	7.5	9	12
Number determinations	201	190	165	152	138	126	118
*Plasma ferritin (µg/L)*							
All	242 ± 125	184 ± 103	88 ± 57	44 ± 29	40 ± 28	26 ± 17	22 ±18
Range	38–752	43–710	10–373	3–137	5–144	4–90	5–137
Female	256 ± 131	201 ± 106	98 ± 58	51 ± 30	44 ± 29	30 ± 18	23 ± 17
Male	227 ± 119	169 ± 99	80 ± 55	39 ± 27	36 ± 27	24 ± 16	22 ± 19
p M *vs.* F	0.105	0.032	0.040	0.015	0.098	0.045	0.715
Number < 10 µg/L (M/F)	0/0	0/0	1/0	6/2	10/1	12/3	9/6
Number < 12 µg/L (M/F)	0/0	0/0	3/0	9/3	14/2	16/7	15/9
*Transferrin receptor (mg/L)*							
All	3.21 ± 0.65	4.49 ± 1.10	6.52 ± 1.12	6.66 ± 1.17	7.05 ± 1.19	7.12 ± 1.41	6.97 ± 1.14
Female	3.08 ± 0.62	4.23 ± 0.97	6.19 ± 0.95	6.28 ± 1.01	6.74 ± 1.07	6.91 ± 1.28	6.94 ± 0.94
Male	3.34 ± 0.66	4.71 ± 1.15	6.79 ± 1.19	6.97 ± 1.21	7.30 ± 1.23	7.23 ± 1.50	6.90 ± 1.29
p M *vs.* F	0.0037	0.0027	0.0006	0.0003	0.005	0.200	0.85
Correl. coeff. PF *vs*. TfR	0.026	−0.182 ^a^	−0.218 ^a^	−0.433 ^a^	−0.360 ^a^	−0.104	−0.135
*Body iron (mg/kg)*							
All	13.7 ± 1.91	11.4 ± 2.32	7.20 ± 2.67	4.60 ± 2.90	3.03 ± 2.91	2.67 ± 2.48	2.12 ± 2.32
Female	14.1 ± 1.70	12.1 ± 2.01	7.94 ± 2.25	5.45 ± 2.57	4.63 ± 2.57	3.27 ± 2.24	2.26 ± 2.18
Male	13.3 ± 2.02	10.9 ± 2.43	6.58 ± 2.84	3.91 ± 2.59	3.33 ± 3.06	2.22 ± 2.58	2.01 ± 2.44
p M *vs.* F	0.0031	0.0004	0.0011	0.0010	0.0091	0.0188	0.579

^a^ correlation statistically significant (*p* < 0.05).

**Figure 3:**
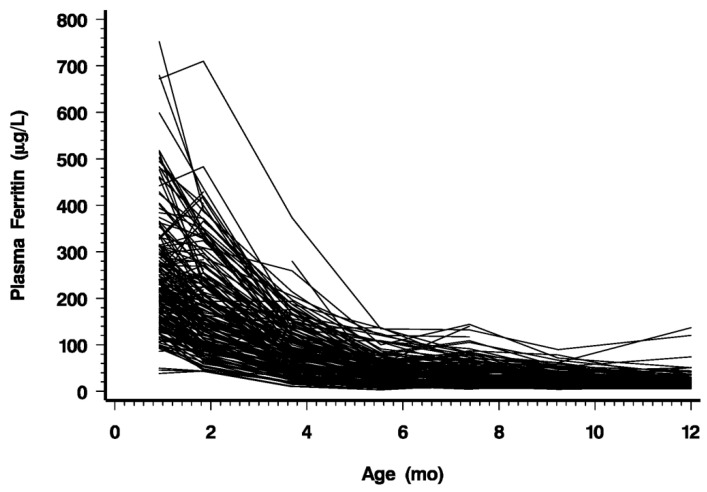
PF of individual infants from 1 to 12 months.

**Figure 4:**
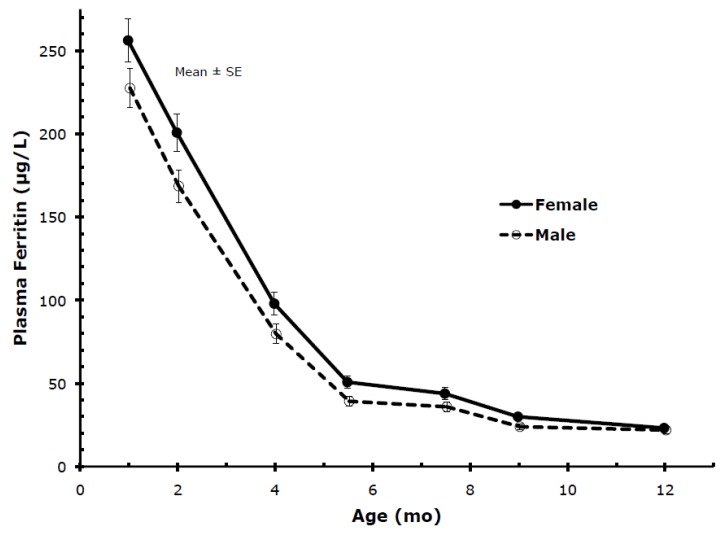
PF of males and females. Differences were statistically significant except at 1 and at 12 months.

As shown in Figure 3 and Table 2, infant PF decreased rapidly with age but leveled off after 5.5 months, indicating exhaustion of the iron endowment. In some infants, PF increased between 1 and 4 months, presumably indicating continuing recycling of iron from hemoglobin breakdown. Transient increases of PF at other ages indicate acute phase reactions. From 2 to 5.5 months, average PF declined by 1.1 (SD 0.40) % each day. The decline was strongly inversely correlated with gain in weight and length (*p* ≤ 0.0001). This is consistent with the notion that growth is the main cause of the decline of PF. As PF decreased, the range of values narrowed progressively. This is illustrated by the percentile values shown in Figure 4. 

Concentrations of TfR increased with age until 7.5 months and then leveled off, mirror- imaging PF values (Table 2). TfR was significantly inversely correlated with PF at most ages. Body iron was highest at 1 month and declined progressively thereafter, in essence paralleling the course of PF.

Tracking: In spite of the marked decrease of PF, there was a strong tendency for infants to preserve their rank. PF values correlated (tracked) strongly over time (Table 3). Although tracking is to be expected given the wide range of PF values at 1 month, tracking continued at 9 and 12 months, which suggests that other factors, including genetic, may be operating. TfR also showed tracking which was less strong than that of PF but was still quite strong (Table 3).

**Table 3:** Pearson correlations among PF (**a**) values and TfR (**b**) values at different ages.

**(a) PF**					
**Age**	**4 months**	**5.5 months**	**7.5 months**	**9 months**	**12 months**
**1 month**	0.670 ^a^	0.638 ^a^	0.496 ^a^	0.546 ^a^	0.465 ^a^
**4 months**	-	0.738 ^a^	0.680 ^a^	0.700 ^a^	0.438 ^a^
**5.5 months**	-	-	0.751 ^a^	0.747 ^a^	0.511 ^a^
**7.5 months**	-	-	-	0.804 ^a^	0.515 ^a^
**9 months**	-	-	-	-	0.579 ^a^
**(b) TfR**					
**Age**	**4 months**	**5.5 months**	**7.5 months**	**9 months**	**12 months**
**1 month**	0.321 ^a^	0.357 ^a^	0.319 ^a^	0.297 ^a^	0.146
**4 months**	-	0.616 ^a^	0.613 ^a^	0.423 ^a^	0.321 ^a^
**5.5 months**	-	-	0.616 ^a^	0.543 ^a^	0.330 ^a^
**7.5 months**	-	-	-	0.605 ^a^	0.515 ^a^
**9 months**	-	-	-	-	0.525 ^a^

^a^ correlation statistically significant (*p*<0.05).

**Figure 5:**
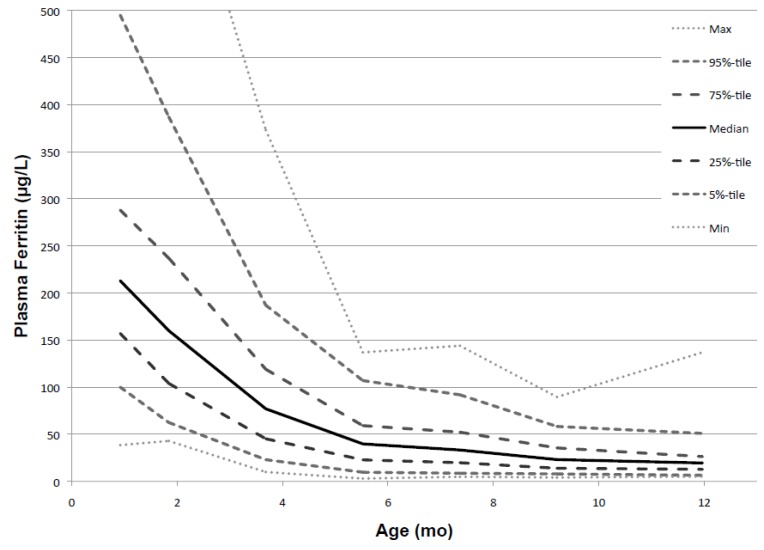
Percentile values for PF from 1 to 12 months (males and females combined).

Gender: Plasma ferritin showed marked gender-related differences as indicated in Table 2 and Figure 5, with levels of female infants being significantly higher than levels of male infants at most ages. Accordingly, female infants became iron deficient (PF < 10 µg/L) less frequently than male infants (Table 2). Gender-related differences were also present in TfR but, contrary to PF, males had higher levels, with differences being statistically significant except at 9 and 12 months. Body iron was significantly higher in females than males except for 12 months.

Infants who developed ID: PF values less than 10 µg/L indicating exhausted iron stores were observed at almost all ages. The earliest age at which PF ≤ 10 µg/L occurred was 4 months in one infant. At 5.5 months, the PF of an additional seven infants dropped below 10 µg/L, meaning that eight (5.3%) infants had exhausted their Fe stores before 6 months. At 28 days, the mean PF of these eight infants was 125 ± 103 µg/L, which was significantly (*p* < 0.001) lower than the PF of all other infants at 28 days (mean 245 ± 119 µg/L). At 7.5 months, an additional nine infants developed a PF of ≤10 µg/L, meaning that 16 (11.6%) infants had exhausted their iron stores. After 7.5 months, with the birth iron endowment exhausted, a PF ≤ 10 µg/L was reflective of low dietary iron intake. This occurred in 15 infants at 9 months and 15 infants at 12 months. A total of 36 infants (19% of those randomized at 4 months) developed a PF of ≤10 µg/L at least once during the study. PF values < 12 µg/L were observed on 78 occasions (Table 2). Among infants with PF ≤ 65 µg/L at 56 days of age (*N* = 12), one developed ID at 4 months and four more by 5.5 months. Thus, among infants with a PF less than 65 µg/L at 2 months, 42% developed ID before 6 months.

When infants developed a PF of ≤10 µg/L, the parents were informed and the suggestion was made by the investigators to increase the consumption of iron-containing foods. One such infant was placed on ferrous sulfate by his pediatrician and subsequent PF values were >10 µg/L. The parents of three infants chose to withdraw from the study. In all remaining infants with ID, PF was monitored closely and in seven infants the next PF was >10 µg/L. In cases where it remained <10 µg/L (*N* = 5) hemoglobin was determined to check for IDA. Hemoglobin was in each case >105 g/100 mL. With IDA ruled out, no iron treatment was recommended. The parents were always informed of findings and feeding of iron-rich foods was recommended whenever PF was ≤10 µg/L.

## 4. Discussion

We believe our cohort to be the largest group of breastfed infants in whom the birth iron endowment was assessed while the intake of iron from exogenous sources was minimal. Thus, the iron endowment was observed in its undisturbed state while not being modified by dietary iron. The size of the iron endowment was assessed by determination of plasma ferritin concentration (PF). At the earliest assessment at one and two months, the iron endowment showed enormous variation. This wide range in the size of the iron endowment has been previously shown by us [4,5,6] and others [7,8,9,10,11,12,13,14,15,16,17,18]. Only a small proportion of that variation was explained by iron status of the mother whereas its bulk remained unexplained. Earlier studies found for the most part no association or only a weak association between maternal iron status and infant iron status at birth [7,9,10,16,17,18]. Some studies did find a reduction of infant iron status when maternal iron status was very poor [8,11,12]. Except for some maternal conditions that are known to reduce the size of the iron endowment [19], the cause of the variation is unknown.

Because infants of the present cohort were not permitted to receive supplemental formula, a source of dietary iron, we expected the iron endowment to be somewhat smaller than in the control infants of our earlier study [5] who received complementary foods like the present cohort but did also receive supplementary formula. However, PF values were nearly identical in that earlier group [5] and in the present cohort. For example, at 5.5 months mean PF in the earlier group was 42 ± 29 µg/L and was 44 ± 29 µg/L in the present study. The explanation may be that infants who did receive formula received only modest amounts, and many infants probably received no formula at all. 

While the cause of the variation in the size of the iron endowment remains obscure, its consequences are readily apparent. The small size of the iron endowment means that it becomes exhausted early and places the infant at risk of ID. We [5,6] and others [19] have before noted this association between size of the iron endowment and risk of ID early in life. In the present cohort a full 5.3% of infants developed ID by 6 months. The incidence is comparable to the incidence observed earlier by us [5,6]. Others have reported somewhat lower [26] and also higher [27] incidences of ID by 6 months. The present estimate of the incidence of ID in breastfed infants who receive only modest amounts of dietary iron is solid thanks to the design of the study. On the other hand, the present study provides no estimates of the incidence of IDA. The reasons are that hemoglobin was not determined with all PF determinations but was determined only when PF remained low in spite of the parents being requested to increase the amount of iron-containing foods. Without that intervention, some infants with ID might have gone on to develop IDA on subsequent assessment. 

The present study shows marked gender-related differences in the size of the iron endowment, with girls having significantly greater size than boys. There was also a gender-related difference in TfR except in the opposite direction, with boys having higher values than girls. Similar gender-related differences have been reported by Hay and colleagues [16,26], Domellöf *et al.* [28] and were seen in our earlier studies [5,6]. PF and TfR were inversely correlated at most ages. Our data clearly show that there are gender-related differences in the size of the iron endowment. This means that whatever is causing gender-related differences at later ages is already operating *in utero*.

We showed strong tracking of PF and TfR that decreases in strength somewhat with increasing age but is still strong at 12 months for PF. Tracking of PF has been reported before by Hay *et al.* [16] and by us [5,6]. Tracking, together with gender-related differences, provides a strong suggestion that the size of the iron endowment and iron status in general are controlled by genetic factors. 

The strengths of the present study are that it involved a large cohort of breastfed infants in whom the intake of dietary iron was kept to a practical minimum, permitting study of the natural course of the birth iron endowment without modification by dietary iron. Another strength was that all observations were made in longitudinal fashion and that detailed dietary information was recorded. 

## 5. Conclusions

It was concluded that the birth iron endowment is highly variable in size and differs significantly between males and females. Its size decreases as iron is used for growth. Some infants develop ID by 6 months of age. A small iron endowment places infants at increased risk of iron deficiency. The fact that PF and TfR track strongly during infancy suggests the operation of genetic factors. 
